# The Mediating Role of Difficulties in Emotion Regulation and Earthquake Stress Coping in the Relationship Between Posttraumatic Cognitive Attribution and Posttraumatic Stress Disorder in Türkiye 2023 Earthquake Survivors

**DOI:** 10.1007/s11126-025-10118-w

**Published:** 2025-01-29

**Authors:** Azmi Çağlar

**Affiliations:** Department of Psychological Counseling, Abant Izzet Baysal University, Bolu, Türkiye

**Keywords:** Posttraumatic cognitive attribution, Posttraumatic stress disorder, Difficulties in emotion regulation, Earthquake stress coping

## Abstract

Natural disasters such as earthquakes leave deep psychological effects on individuals that can lead to posttraumatic stress disorder, and understanding these effects is vital to support psychological recovery processes after trauma. In this context, the aim of this study was to examine the mediating role of emotion regulation difficulties, religious coping, positive reappraisal and seeking social support in the relationship between posttraumatic cognitive attributions and posttraumatic stress disorder in 2023 Kahramanmaraş Pazarcık, Elbistan and Hatay Yayladağı earthquake survivors (*N* = 408). The findings from the multiple mediation analysis showed the indirect effect of posttraumatic cognitive attribution on PTSD through difficulties in emotion regulation, religious coping, positive reappraisal, and seeking social support. Therefore, difficulties in emotion regulation may be a risk factor for PTSD, while religious coping, positive reappraisal and seeking social support may be protective factors. Strategies to reduce difficulties in emotion regulation and to improve religious coping, positive reappraisal and social support seeking in earthquake survivors may be necessary to reduce PTSD that may be caused by the earthquake.

## Introduction

On February 6, 2023, at 04:17 and 13:24 Türkiye time, two earthquakes of magnitude Mw 7.7 and Mw 7.6 occurred with epicenters in Pazarcık and Elbistan districts of Kahramanmaraş province. Subsequently, another earthquake with a magnitude of Mw 6.4 occurred on February 20, 2023 at 20:04 Türkiye time with the epicenter in Yayladağı, Hatay. In total, these earthquakes caused great destruction in 11 provinces close to their epicenters. More than 48,000 people lost their lives as a result of the earthquakes. Around 500,000 buildings were damaged and the region's communications, energy and other important infrastructures were damaged. The total number of earthquake victims was announced as 14,013,196. This number corresponds to 16.4% of the total population of Türkiye [28]. As a result of the earthquakes, more than 48,000 people lost their lives and survivors were exposed to major stress factors such as loss of loved ones, difficulties in accessing daily life needs and shelter.

As a natural disaster, earthquakes cause deaths, physical illnesses, infrastructure damage and economic losses, but they also have long-term mental health effects. Depression, cognitive impairment, panic disorder, and post-traumatic stress disorder (PTSD) are commonly reported as psychological effects [38]. Among these psychological effects, PTSD is one of the psychological disorders that may frequently occur after disasters and various traumatic events [24]. The American Psychiatric Association [APA] ([2] defines PTSD as exposure to death, severe injury, or sexual assault in a real or horrifying way that includes direct exposure to events that individuals themselves have been directly exposed to, direct exposure to events experienced by others, learning that their primary relatives and close friends have been exposed to traumatizing experiences, or repeatedly encountering the details of traumatic events. Based on the definition, it is understood that the number of people exposed to post-earthquake traumatic experiences is not limited to those who were directly exposed, but has a widespread impact. Indeed, research shows that PTSD is the most common mental health problem among earthquake survivors [10]. On the other hand, Tang [34] reported that the prevalence of post-earthquake PTSD ranged between 4.10% and 67.07% in adults. Similarly, in a study conducted by Wang et al. [37] on survivors of the Wenchuan earthquake, PTSD was found to be common. In studies on risk factors for the development of PTSD after the earthquake, significant differences were found between individuals at low risk for PTSD and individuals at high risk for PTSD in terms of cognitive style [21]. Therefore, research shows that non-adaptive cognition related to trauma is among the ultimate predictors of PTSD symptoms [36].

The concept of posttraumatic cognitive attribution, which refers to non-adaptive attributions after a traumatic experience, may play an important role in the emergence and maintenance of PTSD symptoms after a traumatic experience [4]. Posttraumatic cognitive attribution includes the dimensions of perceived loss of control, impaired perception of continuity and self-blame. In order to fulfill the diagnostic criteria for PTSD: (1) continuous re-experiencing of the event (trauma-related memories, nightmares and flashbacks),(2) avoidance symptoms (trauma-related thoughts and feelings); (3) a negative change in general sensitivity (negative affect, feelings of guilt or self-blame about oneself or the world); and (4) increased arousal and reactivity (hypervigilance, irritability or aggression, attention and sleep disturbances) (APA, 2022). This may indicate important relationships with PTSD diagnostic criteria when considered within the scope of the subcomponents of posttraumatic cognitive attribution. Research also shows that posttraumatic stress symptoms are positively associated with both negative cognitions and difficulties in emotion regulation [25, 31, 32].

Difficulties in emotion regulation includes emotion dysregulation, difficulty in regulating negative affect, difficulty in regulating behavior while experiencing negative affect, limited emotional awareness, and lack of acceptance of emotional response [20]. Research provides evidence for the relationship between difficulties in emotion regulation and PTSD. PTSD has been associated with negative emotions such as shame, guilt, anger, and disgust and impairments in the ability to effectively regulate these emotional states. These negative emotions and emotion regulation difficulties are associated with the severity of PTSD resulting from various types of trauma [22]. In addition, studies on trauma survivors have shown that those diagnosed with PTSD have difficulties in emotion regulation, and also showed that emotion regulation difficulties before the traumatic experience predict PTSD symptoms [3, 5, 7, 13]. From this point of view, emotion regulation is stated to be an important variable for PTSD [42], and the importance of coping strategies with the stress caused by the earthquake is also emphasized for PTSD [39].

Earthquake survivors try to cope with the trauma in different ways. Coping is defined as the thoughts and behaviors used to manage the internal and external demands of situations that are considered stressful [12]. Research shows that there are relationships between adaptive and maladaptive coping strategies and PTSD. Adaptive mechanisms include the use of religious, familial and social support, withdrawal and helping others (Adhikari Baral & KC, [1]). On the other hand, it is reported that those with PTSD symptoms often use maladaptive coping strategies in the areas of denial, internalization, behavioral detachment and self-blame to cope with the earthquake [6]. In addition, positive religious coping and active coping are positive predictors of PTSD [23].

The clinical significance of examining the relationship between posttraumatic cognitive attribution (PTCA) and the mediating factors difficulty in emotion regulation, religious coping, positive reappraisal, and seeking social support lies in its potential to inform targeted therapeutic interventions for earthquake survivors. Research suggests that maladaptive cognitive attributions play a critical role in the maintenance of PTSD symptoms, and interventions targeting these attributions can significantly reduce distress [36]. Additionally, addressing mediating factors such as emotion regulation difficulties and coping strategies in trauma-focused therapy may enhance the effectiveness of treatment. For instance, strengthening adaptive coping strategies and improving emotion regulation have been shown to buffer against PTSD symptoms in trauma survivors [22, 39]. By integrating these findings, clinicians can design interventions that simultaneously address cognitive and emotional dimensions of trauma, providing a holistic approach to improving mental health outcomes in earthquake survivors.

Difficulty in emotion regulation may be a risk factor, while religious coping, positive reappraisal and seeking social support may have protective effects on the relationship between posttraumatic cognitive attribution and PTSD. When the literature is examined, there is no study examining the effect of these components on the relationship between posttraumatic cognitive attribution and PTSD in earthquake survivors. However, it is important to understand the mediating roles of these factors. This is because PTCA reflects deeply ingrained, maladaptive cognitive schemas that perpetuate PTSD symptoms. For example, while positive reappraisal may intuitively seem to counteract PTCA, its inclusion in the model highlights the dynamic process by which survivors reframe and reinterpret traumatic experiences. This process is particularly important in contexts where trauma is collective, as in earthquake disasters, and where cultural and community-based coping mechanisms play an important role. Moreover, religious coping, emotion dysregulation and social support are not equally protective in all contexts, and their effects vary depending on the severity of cognitive distortions and the socio-cultural context. In this context, the aim of this study was to investigate the mediating role of difficulty in emotion regulation, religious coping, positive reappraisal and seeking social support in the relationship between posttraumatic cognitive attribution and PTSD in survivors of the 2023 Kahramanmaraş Pazarcık, Elbistan and Hatay Yayladağı earthquakes. Based on the literature, this study has two hypotheses. As the first hypothesis, posttraumatic cognitive attribution will be positively related to PTSD. As the second hypothesis, the relationship between posttraumatic cognitive attribution and PTSD will be mediated by difficulty in emotion regulation, religious coping, positive reappraisal and seeking social support.

## Method

### Participants

The data were collected face-to-face by the researcher seven months after the earthquake. The participants consisted of earthquake survivors who stayed in temporary residences prepared under state protection because their houses were severely damaged after the earthquake. After obtaining permission from the authorized units, the participants were informed about the study and agreed to participate in the study. Of the participants (*N* = 408), 252 (61.8%) were female and 156 (38.2%) were male. The age range of the participants was between 18 and 45 years (Mean = 22.78, SD = 4.98). Of the participants, 340 (83.3%) had experienced at least one bereavement and 68 (16.7%) reported no bereavement.

### Measures

#### Posttraumatic Cognitive Attribution Scale

It was developed by Caglar and Deniz [4]. The scale has 16 items in total. The scale has three sub-dimensions (perceived loss of control, perception of impaired continuity, self-blame) and a 7-point Likert scale (1 = strongly disagree, 7 = strongly agree). The psychometric properties of the scale show that it has a valid structure (x^2^/df = 2.62, TLI = 0.91, CFI = 0.93, GFI = 0.93, IFI = 0.93, RMSEA = 0.06, SRMR = 0.03). The lowest score that can be obtained from the scale is 16 and the highest score is 112. High scores on the scale indicate non-adaptive cognitive attributions after trauma. Reliability analysis of the scale (α = 0.86) shows that it has an adequate internal consistency coefficient.

#### Posttraumatic Stress Disorder Short Scale

The National Stressful Events Survey PTSD Short Scale, developed by LeBeau et al. [19], is based on DSM-5 diagnostic criteria and measures cognitive-emotional reactions including withdrawal, avoidance, and hyper-vigilance. The scale consists of a 4-point Likert scale (0 = not at all, 4 = extremely) and 9 items, with a minimum score of 0 and a maximum score of 36. Reliability analysis of the scale (α = 0.89) shows that it has an adequate internal consistency coefficient. In addition, a clinical cut-off score was calculated over 24 points. (2016) reported that the scale, which was adapted into Turkish by Evren et al. [9], is a psychometrically valid PTSD screening measure with high discriminant and convergent validity (*r* = 0.79) and reliability (α = 0.87).

#### Difficulty in Emotion Regulation Scale

The Turkish adaptation study of the *Difficulty in Emotion Regulation Scale*−8 developed by Penner et al. (2022) was conducted by Ekşi and Erik [8]. The scale, which explains emotion dysregulation with fewer items, consists of four sub-dimensions (goal, impulse, nonacceptance, strategy) and eight items. The scale has a 5-point Likert scale (0%−10% = Almost never, 91%−100% = Almost always). The internal consistency value for the whole scale was found to be α = 0.87. Construct validity goodness of fit index values (x^*2*^/df = 3.05, NFI = 0.96, CFI = 0.97, TLI = 0.95, RMSEA = 0.07) were found to be at an acceptable level.

#### Earthquake Stress Coping Scale

Earthquake Stress Coping Scale developed by Yöndem and Eren [40] measures the coping strategies of individuals with earthquake stress. The scale has 16 items in total. The scale has three subscales (religious coping, positive reappraisal, seeking social support) and a 4-point Likert scale (1 = never, 4 = always). The psychometric properties of the scale show that it has a valid structure (x^2^/df = 1.45, CFI = 0.94, NNFI = 0.93, RMSEA = 0.049). Higher scores on the religious coping (lowest score 5—highest score 20), positive reappraisal (lowest score 6—highest score 24), and social support seeking (lowest score 5—highest score 20) scales indicate higher levels of religious coping, positive reappraisal, and social support seeking strategies.

### Data Analysis

Statistical assumptions, descriptive statistics and correlations between variables were calculated (*p* < 0.01). In addition, kurtosis and skewness values were calculated to test the normality of the data [33]. The relationships between the study variables were calculated using the Pearson Correlation Analysis technique and then the mediation analysis was calculated using the Process macro for the SPSS version. Process macro mediation analysis is used to calculate direct and indirect relationships between the study variables through multiple or single mediators [15]. In addition, different reliability coefficients (Cronbach's alpha (α), McDonald's omega (ω), Guttmann's lambda (λ6)) were evaluated. For the mediation model, standard path coefficient values (β) were calculated between the study variables. In addition, 5000 bootstrap samples were used for the significance of indirect effects (95% CI). A bootstrap confidence interval greater than or less than zero for indirect effects indicates that the mediation effect is significant [15]. Analyses were calculated using SPSS version 26.

## Results

Table 1 shows a significant positive correlation between posttraumatic cognitive attribution and posttraumatic stress disorder (Results confirmed hypothesis 1). There was a positive correlation between posttraumatic cognitive attribution and posttraumatic stress disorder and difficulty in emotion regulation. Significant negative correlations were found between posttraumatic cognitive attribution and PTSD and religious coping, positive reappraisal and seeking social support. Skewness and kurtosis values were within the acceptable range and the data were normally distributed [33]. Reliability analyses were also found to be within the acceptable range.

**Table 1:** Correlations and descriptive statistics (*N* = 408)

Variables	1	2	3	4	5	6
1. PTCA	1					
2. PTSD	.516**	1				
3. DER	.240**	.654**	1			
4. RC	-.287**	-.317**	-.182**	1		
5. PR	-.382**	-.541**	-.193**	.502**	1	
6. SSS	-.330**	-.581**	-.213**	.742**	.578**	1
*M*	54.65	16.02	27.95	9.81	12.93	10.54
*SD*	10.54	11.13	4.27	2.31	3.77	2.56
Skewness	0.238	-.285	-.269	.244	.472	.316
Kurtosis	1.45	-.1.44	-.211	-.556	-.282	.595
Cronbach alfa (α)	.807	.977	.903	.873	.896	.701
McDonald's omega (ω)	.769	.977	.904	.874	.899	.703
Guttmann's lambda (λ6)	.901	.977	.910	.916	.890	.762

^**^p < .01 *(PTSD: posttraumatic stress disorder; PTCA: posttraumatic cognitive attribution; DER: difficulties in emotion regulation; RC: religious coping; SSS: seeking social support; PR: positive reappraisal)*

### Mediational Analyses

**Fig. 1:**
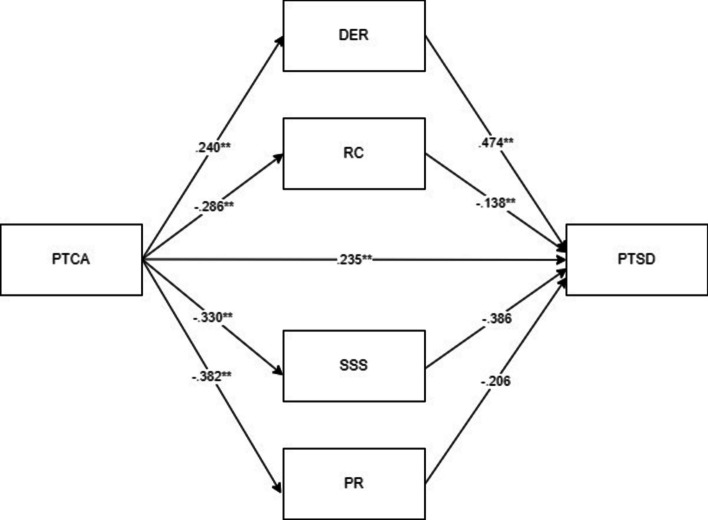
Parallel multiple mediation effect of difficulties in emotion regulation, religious coping, positive reappraisal and seeking social support in the relationship between posttraumatic cognitive attribution and posttraumatic stress disorder. **p < .001 (*Note. PTSD: posttraumatic stress disorder; PTCA: posttraumatic cognitive attribution; DER: difficulties in emotion regulation; RC: religious coping; SSS: seeking social support; PR: positive reappraisal)*

The findings regarding the role of parallel mediating variables in the relationship between posttraumatic cognitive attribution and PTSD are given in Fig. 1.

The direct effect of posttraumatic cognitive attribution (β = 0.235, *p* < 0.001) on PTSD was statistically significant. The results also show that all simple path mediation coefficients, difficulty in emotion regulation (β = 0.240, *p* < 0.001), religious coping (β =—0.286, *p* < 0.001), seeking social support (β =—0.330, *p* < 0.001), and positive reappraisal (β =—0.382, *p* < 0.001) were statistically significant. Finally, the effects of difficulty in emotion regulation (β = 0.474, *p* < 0.001), religious coping (β =—0.138, *p* < 0.001), seeking social support (β =—0.386, *p* < 0.001), and positive reappraisal (β =—0.206, *p* < 0.001) on PTSD were also significant.

Table 2 presents the findings on the indirect effects of the four mediating variables. Standardized Beta coefficients are given (95% CI). Accordingly, posttraumatic cognitive attribution has an indirect effect on PTSD through difficulty in emotion regulation (β = 0.114, SE = 0.019, *p* < 0.001), religious coping (β = 0.069, SE = 0.021,* p* < 0.001), positive reappraisal (β = 0.078, SE = 0.013, *p* < 0.001), and seeking social support (β = 0.127, SE = 0.016, *p* < 0.001). In conclusion, the results also confirmed hypothesis 2.

**Table 2:** The indirect effect of posttraumatic cognitive attribution on PTSD through emotion dysregulation, religious coping, positive reappraisal, and seeking social support

Path	Indirect effects	*SE*	Boot LLCI	Boot ULCI
PTCA → DER → PTSD	.114	.015	.083	.144
PTCA → RC → PTSD	.069	.012	.065	.113
PTCA → PR → PTSD	.078	.013	.053	.107
PTCA → SSS → PTSD	.127	.016	.096	.162

*(PTSD: posttraumatic stress disorder; PTCA: posttraumatic cognitive attribution; DER: difficulties in emotion regulation; RC: religious coping; SSS: seeking social support; PR: positive reappraisal)*

## Discussion

The aim of this study was to investigate the mediating role of difficulty in emotion regulation, religious coping, positive reappraisal and seeking social support in the relationship between posttraumatic cognitive attribution and PTSD in survivors of the 2023 Kahramanmaraş Pazarcık, Elbistan and Hatay Yayladağı earthquakes. The findings of the study show that there is a positive correlation between posttraumatic cognitive attribution and PTSD. In addition, the results show the indirect effect of posttraumatic cognitive attribution on PTSD through difficulty in emotion regulation, religious coping, positive reappraisal, and seeking social support. These findings suggest that difficulty in emotion regulation may contribute as a risk factor for PTSD among earthquake survivors, while religious coping, positive reappraisal, and social support seeking may potentially contribute as protective factors.

The finding that posttraumatic cognitive attribution is positively associated with PTSD is supported by previous research. Kannis-Dymand et al. [18] investigated the cognitive themes that developed in those differently affected by the Christchurch earthquake and aftershocks. The results show that negative cognitions about the earthquake are significantly associated with depression, anxiety and stress [17] as well as acute stress disorder. In a 2011 study on posttraumatic negative cognitions among those affected by the Great East Japan Earthquake, it was reported that negative cognitions were associated with high levels of PTSD [29]. Similarly, it was stated that cognitive factors related to trauma are among the risk factors for PTSD [27]. These findings emphasise the effect of posttraumatic cognitive attributions on PTSD and show that negative cognitions are an important factor shaping individuals' psychological responses to trauma. In this context, identifying and transforming negative posttraumatic cognitions may offer a critical intervention area for the prevention or support of PTSD in individuals exposed to disasters such as earthquakes.

The findings also showed that difficulty in emotion regulation, religious coping, positive reappraisal, and seeking social support partially mediated the relationship between posttraumatic cognitive attribution and PTSD. Research has emphasized the protective role of adaptive coping strategies in studies focusing on earthquake survivors. Indeed, Adhikari Baral and KC [] state that maladaptive coping strategies increase the likelihood of PTSD and promoting adaptive coping strategies is a protective factor for PTSD. On the other hand, Zhang et al. [41] reported that maladaptive coping strategies are a strong risk factor for PTSD symptoms. A study conducted on survivors 2 years after the 2017 Jiuzhaigou earthquake suggests that adaptive coping strategies may help them cope with stress after the earthquake [35]. In addition, Huang et al. [16] stated that low social support is among the risk factors for PTSD. In a study conducted with Pakistan earthquake survivors, negative religious coping was associated with higher PTSD symptom levels and negative emotions. In addition, higher perceived social support was associated with higher positive emotions [11]. Considering that negative religious coping is the state of feeling punished by God for one's sins or lack of spirituality, it can be said that whether the religious coping strategies of earthquake survivors are positive or negative may be an effective factor in being a protective or risk factor for PTSD. In addition to adaptive coping strategies being a protective factor for PTSD, studies have emphasized that difficulty in emotion regulation is a risk factor for PTSD. It is emphasized that PTSD symptom severity is significantly related to difficulty in emotion regulation [7]. It was also reported that individuals diagnosed with PTSD reported more difficulty in emotion regulation [14]. Again, PTSD causes difficulty in regulating especially negative emotions [30]. These findings emphasise the importance of both risk and protective factors in the development of PTSD. In particular, emotion regulation difficulties seem to be a critical risk factor that increases PTSD, whereas adaptive coping strategies such as religious coping, positive reappraisal and seeking social support play a protective role. In this context, developing adaptive coping strategies and strengthening emotion regulation skills in individuals exposed to traumatic events such as earthquakes can be considered as an effective intervention strategy to reduce the risk of PTSD.

## Conclusion

In conclusion, research findings show that posttraumatic cognitive attribution variable is significantly positively associated with PTSD. Non-adaptive cognitive attributions developed for the earthquake may increase the level of PTSD. PTSD is significantly negatively associated with religious coping, positive reappraisal and seeking social support. Therefore, earthquake survivors with high levels of religious coping, positive reappraisal and social support seeking can cope with the stress that the earthquake may cause. Finally, PTSD is significantly positively associated with difficulty in emotion regulation. Therefore, it can be said that those who have difficulty in emotion regulation are more vulnerable to develop PTSD.

## Limitations and Future Research

The study findings should be considered in the context of several limitations. First, the current study is a cross-sectional study. Therefore, future longitudinal studies may investigate the underlying causes of PTSD, difficulty in emotion regulation, religious coping, positive reappraisal and seeking social support. Secondly, the data were collected from those who were directly exposed to the earthquake. However, the 2023 Kahramanmaraş Pazarcık, Elbistan, and Hatay Yayladağı earthquakes affected a wide geography in Türkiye as well as the people of the region. People in other parts of Türkiye, which is considered an earthquake zone, also experienced the anxiety of facing a similar earthquake. Therefore, similar studies can be conducted with a larger sample. Finally, this study was conducted in Türkiye and similar research can be conducted in other countries. It is also recommended that clinical practitioners adopt some approaches in the light of research findings when working with individuals exposed to post-earthquake trauma. Psychotherapeutic interventions aimed at improving individuals' ability to recognise, understand and regulate their emotional reactions can be integrated with trauma-focused therapy approaches, especially due to the positive association of emotion regulation difficulties with PTSD. Moreover, promoting adaptive coping strategies such as religious coping, positive reappraisal, and seeking social support may help individuals cope more effectively with posttraumatic stress. In this context, psychosocial support programmes that support individuals' religious beliefs and encourage positive coping methods can be established. In addition, in order to increase the protective effect of seeking social support on PTSD, group therapies or community-based interventions can be implemented to strengthen the family, friend and community support networks of earthquake survivors.

## Data Availability

Data will be available on request.

## Declarations

### Pre-registration Statement

This study was not pre-registered.
